# Chromosomal microarray analysis as the first-tier test for the identification of pathogenic copy number variants in chromosome 9 pericentric regions and its challenge

**DOI:** 10.1186/s13039-016-0272-6

**Published:** 2016-08-10

**Authors:** Jia-Chi Wang, Fatih Z. Boyar

**Affiliations:** Cytogenetics Laboratory, Quest Diagnostics Nichols Institute, 33608 Ortega Highway, San Juan Capistrano, CA 92690 USA

**Keywords:** Chromosome 9, Euchromatic variants, First-tier test, Heterochromatic variants, Interstitial deletion, Interstitial duplication, Pericentric inversion

## Abstract

Chromosomal microarray analysis (CMA) has been recommended and practiced routinely in the large reference laboratories of U.S.A. as the first-tier test for the postnatal evaluation of individuals with intellectual disability, autism spectrum disorders, and/or multiple congenital anomalies. Using CMA as a diagnostic tool and without a routine setting of fluorescence in situ hybridization with labeled bacterial artificial chromosome probes (BAC-FISH) in the large reference laboratories becomes a challenge in the characterization of chromosome 9 pericentric region. This region has a very complex genomic structure and contains a variety of heterochromatic and euchromatic polymorphic variants. These variants were usually studied by G-banding, C-banding and BAC-FISH analysis. Chromosomal microarray analysis (CMA) was not recommended since it may lead to false positive results. Here, we presented a cohort of four cases, in which high-resolution CMA was used as the first-tier test or simultaneously with G-banding analysis on the proband to identify pathogenic copy number variants (CNVs) in the whole genome. CMA revealed large pathogenic CNVs from chromosome 9 in 3 cases which also revealed different G-banding patterns between the two chromosome 9 homologues. Although we demonstrated that high-resolution CMA played an important role in the identification of pathogenic copy number variants in chromosome 9 pericentric regions, the lack of BAC-FISH analysis or other useful tools renders significant challenges in the characterization of chromosome 9 pericentric regions.

**Trial registration:** None; it is not a clinical trial, and the cases were retrospectively collected and analyzed.

**Electronic supplementary material:**

The online version of this article (doi:10.1186/s13039-016-0272-6) contains supplementary material, which is available to authorized users.

## Headings

CMA as the first-tier test for the identification of pathogenic CNVs in chromosome 9 pericentric regions and its challenge.

## Letter to the Editors

Chromosomal microarray analysis (CMA) has been recommended as the first-tier test for the postnatal evaluation of individuals with intellectual disability, autism spectrum disorders, and/or multiple congenital anomalies since 2010 [1, 2], and was later confirmed by other group [3]. This practice has become a standard for the large reference laboratories in U.S.A after the American College of Medical Genetics (ACMG) published the professional guidelines [4]. In these laboratories, CMA has largely replaced fluorescence in situ hybridization (FISH) analysis for the identification of pathogenic copy number variants (CNVs) across the whole genome.

Chromosome 9 pericentric regions have been known to contain a very complex genomic structure because of the presence of a huge amount of heterochromatin and large tracks of segmentally duplicated euchromatin [5, 6]. The segmentally duplicated sequences predispose and mediate the generation of deletion, duplication, insertion, triplication and amplification variants within the pericentric regions of chromosome 9 [5, 7]. In a cohort of 334 carriers studied by using FISH analysis with different sets of labeled bacterial artificial chromosome (BAC) probes (BAC-FISH), 17 different types of heterochromatic variants of chromosome 9 have been identified [6], with pericentric inversions being the most frequent variant (50 %) followed by 9qh-variants (24 %) and 9ph-variants (11 %). Additionally, four different types of euchromatic variants have been detected: (1) 9p12 amplification variant [8, 9], (2) 9q12 insertion variant [5], (3) 9q21 deletion variant [5], and (4) 9q21 amplification variant [7]. Because of the presence of repetitive sequences, chromosomal microarray analysis (CMA) was not recommended to be utilized since it may lead to false positive results [6, 7].

For this reason, we retrospectively reviewed our postnatal database to search interesting cases with CNVs in chromosome 9. High-resolution CMA was either used as the first-tier test or simultaneously ordered with G-banding analysis. For G-banding, peripheral blood was cultured for 72 h in RPMI-1640 medium. Metaphase chromosomes were analyzed using standard G-banding techniques. CMA was performed using genomic DNA extracted from uncultured whole blood on the oligo-SNP array (CytoScan HD^®^, Affymetrix). Hybridization, data extraction and analysis were performed as per manufacturer’s protocols. The Affymetrix^®^ Chromosome Analysis Suite (ChAS) Software version 2.0 was used for data analysis, review and reporting. For chromosome 9, the probes from CytoScan HD^®^ covered the following genomic regions: 9p (chr9:192,129–40,784,142, 43,400,082–44,900,526) and 9q (chr9:66,837,485–141,025,328). Thresholds are set at >200 kb for gains, >50 kb for losses for genome-wide region, and at >100 kb for gains, >20 kb for losses for cytogenetic relevant regions. Genomic coordinates are based upon genome build 37/hg19 (2009).

**Fig. 1:**
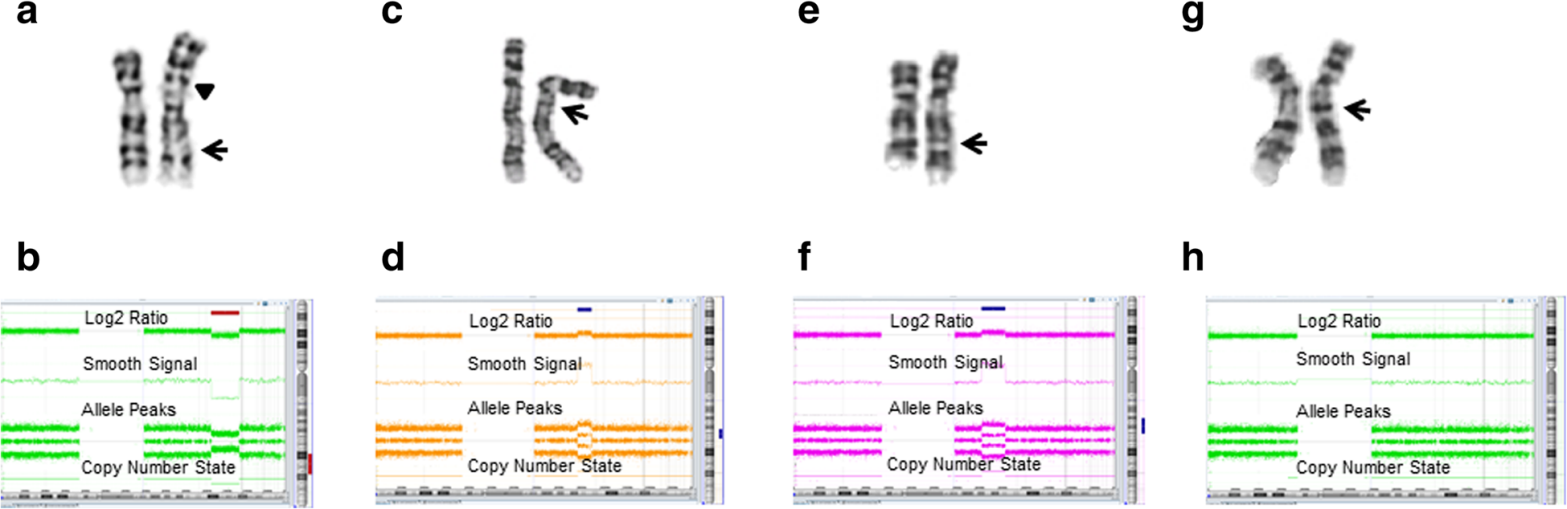
**a** G-banding analysis of patient 1 revealed a pericentric inversion of chromosome 9; the missing band 9q31 (*arrow*) was initially considered to be inverted to 9p13 region (*arrow head*); **b** CMA identified a pathogenic interstitial deletion of 14 Mb at 9q22.3-q32 (chr9:104,382,544–118,273,644; hg19); **c** G-banding analysis of patient 2 revealed an extra band at 9qh; **d** CMA identified a pathogenic gain of 6.3 Mb from 9q21.33–q22.31 (chr9:90,118,500–96,395,801); **e** G-banding analysis of patient 3 revealed an extra band at the 9q22.1 region; **f** CMA identified a 10.4 Mb pathogenic gain from 9q21.31–q22.2 (chr9:82,745,056–93,173,691); **g** G-banding analysis of patient 4 revealed an extra band at 9q12 region with a morphology similar to that of patient 2; **h** CMA did not reveal copy number changes, which ruled out the presence of pathogenic variants

Patient 1 was a newborn girl who was referred to rule out a chromosomal anomaly. Using G-banding, it was initially reported as a common chromosome 9 normal pericentric inversion variant, 46,XX,inv(9)(p13q21.11), with a morphology similar to inv(9)(var2) in the previous report [6]. The second reviewer thought it was a large pericentric inversion, 46,XX,inv(9)(p13q32) since the band 9q31 was missing (Fig. 1a, arrow) and instead inverted to 9p13 region (Fig. 1a, arrow head), and considered that it was not a variant. Further characterization of this large pericentric inversion by concurrent high-resolution CMA revealed the presence of an interstitial deletion of 14 Mb at 9q22.3–q32 (chr9:104,382,544–118,273,644; hg19; Fig. 1b; the genes involved were listed in the Additional file 1: Table S1). The revised karyotype was 46,XX,del(9)(q22.3q32)inv(9)(p13q21.11). It was concluded that two chromosomal rearrangements have occurred to the same chromosome 9: a polymorphic pericentric inversion and an interstitial deletion of 9q22.3–q32. It is actually very difficult to distinguish between the deletion of 9q22–q32 and 9q32–q34 without using molecular cytogenetic methods [10, 11]. Usually the patients with interstitial deletions involving 9q22 had a loss of the *PTCH1* gene (chr9: 98,205,265–98,279,247), and thus revealed typical features of Gorlin syndrome [12–14]. However, since the deletion in this patient was distal to the *PTCH1* gene, this newborn girl did not have Gorlin syndrome.

Patient 2 was a 2-year-old girl with developmental delay, lack of coordination, mixed receptive expressive language disorder and convulsion. It was initially considered as a chromosome 9 euchromatic variant with an extra G-dark band within the proximal 9q heterochromatin region or it could be an insertion or duplication from 9q13–q21.1 (Fig. 1c, arrow). The C-banding was negative. This type of euchromatic variant has been reported in several cases [5, 15, 16] and classified as one of the four major categories of euchromatic variant [7]. The high-resolution CAM that was simultaneously performed, however, showed a genomic gain of 6.3 Mb from 9q21.33–q22.31 (chr9:90,118,500–96,395,801; hg19; Fig. 1d; genes involved listed in the Additional file 1: Table S1). This case was an excellent example of how CMA analysis showed that an extra G-dark band in proximal 9q was a pathogenic duplication of proximal 9q rather than a euchromatic variant. This gain did not include the *PTCH1* gene (located at chr9: 98,205,265–98,279,247) and, thus the phenotype was different from the previously reported familial cases with 9q22.3 microduplication spanning *PTCH1* [17], and without microcephaly or holoprosencephaly that were often seen in patients with gain of *PTCH1* [18].

Patient 3 was a 24-year-old woman referred to rule out a chromosomal disorder with reported family history of a chromosome 9 abnormality. G-banding analysis considered it was an insertion of an extra band to the 9q22 region (Fig. 1e). CMA revealed it was a duplication of 10.4 Mb from 9q21.31-q22.2 (chr9:82,745,056–93,173,691; Fig. 1f; genes involved listed in the Additional file 1: Table S1). Duplication of 9q21.2–q22.3 was reported in a 2-year-old female with growth and motor retardation and in her aunt without apparent phenotypic anomalies [19], and a more distal duplication of 9q22.31–q22.32 was associated with congenital diaphragmatic hernia [20]. Our patient was not known to have these anomalies.

Patient 4 was a 10-year-old boy with failure to thrive and cognitive deficits. G-banding analysis revealed an extra band at 9q12 region (Fig. 1g), and C-banding was positive (Additional file 2: Figure S1). CMA did not reveal copy number variants (Fig. 1h) since the probes from the CMA apparently did not cover the repetitive sequences. The G-positive and C-positive extra band in this case was concluded as a rare heterochromatic variant [6], instead of a G-positive and C-negative euchromatic variant [7].

The four cases described here demonstrate how CMA characterize the chromosome 9 pericentric regions along with G-banding and/or C-banding without access to BAC-FISH analysis. Patient 1 was a very rare case with the presence of an interstitial deletion which coexisted with a polymorphic pericentric inversion. Patient 2 was an excellent example of how CMA differentiated a pathogenic gain from a heterochromatic variant as observed in patient 4. In patient 3, CMA revealed it was a likely pathogenic duplication although previous report showed a reduced penetrance in one family study [19].

Although we demonstrated that high-resolution CMA played an important role in the identification of pathogenic copy number variants in chromosome 9 pericentric regions, the lack of BAC-FISH analysis as a routine setting in the large reference laboratory renders significant challenges in further characterization of chromosome 9 variants. The major advantage of using CMA as the first-tier test is to detect pathogenic CNVs across the whole genome including chromosome 9. However, when a huge amount of heterochromatin and large tracks of segmentally duplicated euchromatin are present in chromosome 9 pericentric regions, CMA was unable to differentiate tandem, inverted and insertional duplication, or to confirm pericentric inversion. Our cohort supported using CMA as first-tier test when chromosomal disorders were indicated to rule out the presence of pathogenic copy number variants, and using BAC-FISH analysis to further evaluate heterochromatic and euchromatic variants of chromosome 9.

## Abbreviations

BAC, bacterial artificial chromosome; CMA, chromosomal microarray analysis; FISH, fluorescence in situ hybridization

## Additional files

Additional file 1: Table S1. List of genes that are involved in the CNVs of case 1-3. (DOC 32 kb) Additional file 2: Figure S1. The extra G-positive band was most likely C-positive by C-banding analysis (arrow) in one chromosome 9 homologue, which indicated that the extra band was heterochromatin in origin. The other chromosome 9 homologue also showed a large amount of heterochromatin (arrow head). The C-banding result was not 100 % conclusive, and thus using BAC-FISH with proper probes will be able to confirm this result. (JPG 28 kb)
