# Supplementary material for: Chromosomal microarray analysis as the first-tier test for the identification of pathogenic copy number variants in chromosome 9 pericentric regions and its challenge
Source: Mol Cytogenet. 2016 Aug 10;9:64. doi: 10.1186/s13039-016-0272-6 (PMC4980801; doi:10.1186/s13039-016-0272-6)
Supplement: Additional file 1: Table S1. — List of genes that are involved in the CNVs of case 1-3. (DOC 32 kb) [file 13039_2016_272_MOESM1_ESM.doc]

SUPPLEMENTARY TABLE 1

| Case | ISCN | All the genes | OMIM genes |
| --- | --- | --- | --- |
| 1 | arr[hg19] 9q31.1q33.1(104,382,544-118,273,644)x1 | *GRIN3A, CYLC2, SMC2, OR13F1, OR13C4, OR13C3, OR13C8, OR13C5, OR13C2, OR13C9, OR13D1, NIPSNAP3A, NIPSNAP3B, LOC286367, ABCA1, SLC44A1, FSD1L, FKTN, TAL2, TMEM38B, ZNF462, MIR548Q, RAD23B, KLF4, ACTL7B, ACTL7A, IKBKAP, FAM206A, CTNNAL1, TMEM245, MIR32, FRRS1L, EPB41L4B, PTPN3, PALM2, PALM2-AKAP2, AKAP2, C9orf152, TXN, TXNDC8, SVEP1, MUSK, LPAR1, OR2K2, KIAA0368, ZNF483, PTGR1, LRRC37A5P, DNAJC25, DNAJC25-GNG10, GNG10, C9orf84, UGCG, MIR4668, MIR3134, SUSD1, PTBP3, HSDL2, KIAA1958, INIP, SNX30, SLC46A2, ZNF883, ZFP37, FAM225B, LINC00256A, SLC31A2, FKBP15, SLC31A1, CDC26, PRPF4, RNF183, WDR31, BSPRY, HDHD3, ALAD, POLE3, C9orf43, RGS3, ZNF618, AMBP, KIF12, COL27A1, MIR455, ORM1, ORM2, AKNA, DFNB31, ATP6V1G1, C9orf91, LOC100505478, TNFSF15, TNFSF8, TNC, DEC1* | *GRIN3A (606650), CYLC2 (604035), SMC2 (605576), NIPSNAP3A (608871), NIPSNAP3B (608872), ABCA1 (600046), SLC44A1 (606105), FSD1L (609829), FKTN (607440), TAL2 (186855), TMEM38B (611236), RAD23B (600062), KLF4 (602253), ACTL7B (604304), ACTL7A (604303), IKBKAP (603722), CTNNAL1 (604785), MIR32 (609355), FRRS1L (604574), EPB41L4B (610340), PTPN3 (176877), AKAP2 (604582), TXN (187700), SVEP1 (611691), MUSK (601296), LPAR1 (602282), PTGR1 (601274), GNG10 (604389), UGCG (602874), PTBP3 (607527), INIP (613273), SLC46A2 (608956), ZFP37 (602951), SLC31A2 (603088), SLC31A1 (603085), CDC26 (614533), PRPF4 (607795), ALAD (125270), POLE3 (607267), RGS3 (602189), AMBP (176870), KIF12 (611278), COL27A1 (608461), ORM1 (138600), ORM2 (138610), AKNA (605729), DFNB31 (607928), ATP6V1G1 (607296), TNFSF15 (604052), TNFSF8 (603875), TNC (187380), DEC1 (604767)* |
| 2 | arr[hg19] 9q21.33q22.31(90,118,500-96,395,801)x3 | *DAPK1, CTSL1, CTSL3, CTSL1P8, LOC392364, FAM75E1, FAM75C1, CDK20, FAM75C2, SPIN1, NXNL2, LOC286238, MIR4289, C9orf47, S1PR3, SHC3, CKS2, MIR3153, SECISBP2, SEMA4D, GADD45G, UNQ6494, LOC286370, MIR4290, LOC340515, DIRAS2, SYK, LOC100129316, AUH, NFIL3, MIR3910-1, MIR3910-2, ROR2, SPTLC1, LOC100128076, LINC00475, IARS, MIR3651, SNORA84, NOL8, CENPP, OGN, OMD, ASPN, ECM2, MIR4670, IPPK, LOC100128361, BICD2, ANKRD19P, ZNF484, FGD3, SUSD3, C9orf89, NINJ1, WNK2, C9orf129, FAM120AOS, FAM120A, PHF2* | *DAPK1 (600831), CTSL1 (116880), CDK20 (610076), SPIN1 (609936), S1PR3 (601965), SHC3 (605263), CKS2 (116901), SECISBP2 (607693), SEMA4D (601866), GADD45G (604949), DIRAS2 (607863), SYK (600085), AUH (600529), NFIL3 (605327), ROR2 (602337), SPTLC1 (605712), IARS (600709), NOL8 (611534), CENPP (611505), OGN (602383), ASPN (608135), ECM2 (603479), BICD2 (609797), NINJ1 (602062), WNK2 (606249), FAM120A (612265), PHF2 (604351)* |
| 3 | arr[hg19] 9q21.31q22.2(82,745,056-93,173,691)x3 | *TLE1, FAM75D5, FAM75D4, FAM75D3, FAM75D1, RASEF, FRMD3, IDNK, UBQLN1, GKAP1, KIF27, C9orf64, HNRNPK, MIR7-1, RMI1, SLC28A3, NTRK2, AGTPBP1, LOC389765, NAA35, GOLM1, C9orf153, ISCA1, ZCCHC6, GAS1, LOC100506834, LOC440173, LOC494127, C9orf170, DAPK1, CTSL1, CTSL3, CTSL1P8, LOC392364, FAM75E1, FAM75C1, CDK20, FAM75C2, SPIN1, NXNL2, LOC286238, MIR4289, C9orf47, S1PR3, SHC3, CKS2, MIR3153, SECISBP2, SEMA4D, GADD45G, UNQ6494, LOC286370, MIR4290* | *TLE1 (600189), RASEF (611344), FRMD3 (607619), IDNK (611343), UBQLN1 (605046), GKAP1 (611356), KIF27 (611253), C9orf64 (611342), HNRNPK (600712), RMI1 (610404), SLC28A3 (608269), NTRK2 (600456), AGTPBP1 (606830), GOLM1 (606804), ISCA1 (611006), GAS1 (139185), DAPK1 (600831), CTSL1 (116880), CDK20 (610076), SPIN1 (609936), S1PR3 (601965), SHC3 (605263), CKS2 (116901), SECISBP2 (607693), SEMA4D (601866), GADD45G (604949)* |
