# Supplementary figures and images for: Chromosomal microarray analysis as the first-tier test for the identification of pathogenic copy number variants in chromosome 9 pericentric regions and its challenge
Source: Mol Cytogenet. 2016 Aug 10;9:64. doi: 10.1186/s13039-016-0272-6 (PMC4980801; doi:10.1186/s13039-016-0272-6)

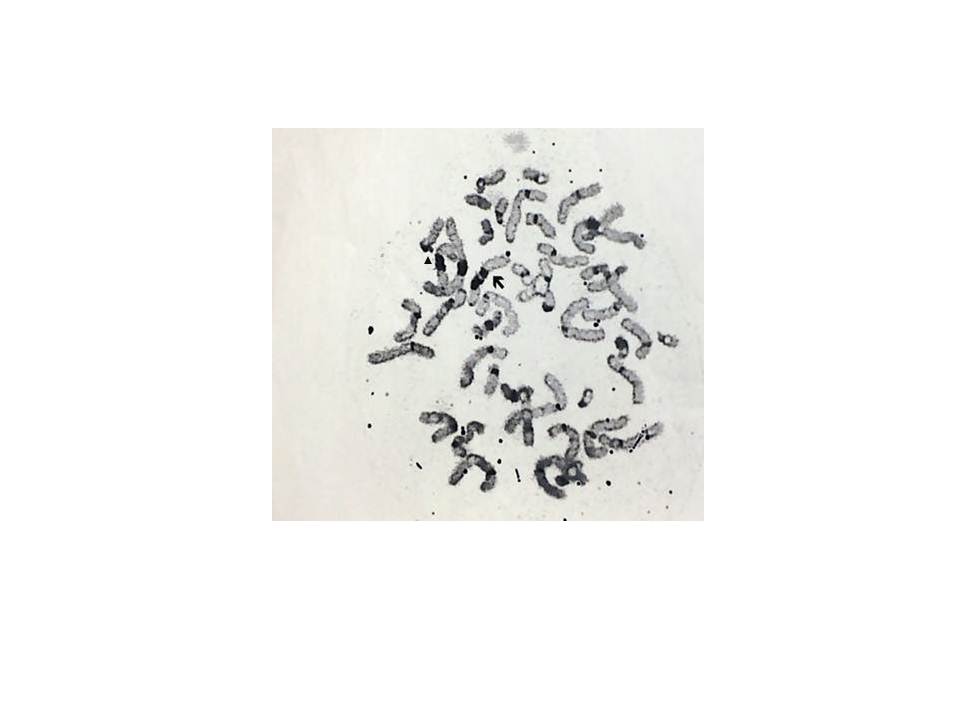

Supplement: Additional file 2: Figure S1. — The extra G-positive band was most likely C-positive by C-banding analysis (arrow) in one chromosome 9 homologue, which indicated that the extra band was heterochromatin in origin. The other chromosome 9 homologue also showed a large amount of heterochromatin (arrow head). The C-banding result was not 100 % conclusive, and thus using BAC-FISH with proper probes will be able to confirm this result. (JPG 28 kb) [file 13039_2016_272_MOESM2_ESM.jpg]
